# Supplementary material for: Concurrent Cetuximab and Nivolumab as a Second-Line or beyond Treatment of Patients with Recurrent and/or Metastatic Head and Neck Squamous Cell Carcinoma: Results of Phase I/II Study
Source: Cancers (Basel). 2021 Mar 9;13(5):1180. doi: 10.3390/cancers13051180 (PMC7967147; doi:10.3390/cancers13051180)
Supplement: Supplementary file 1 [file cancers-13-01180-s001.pdf]

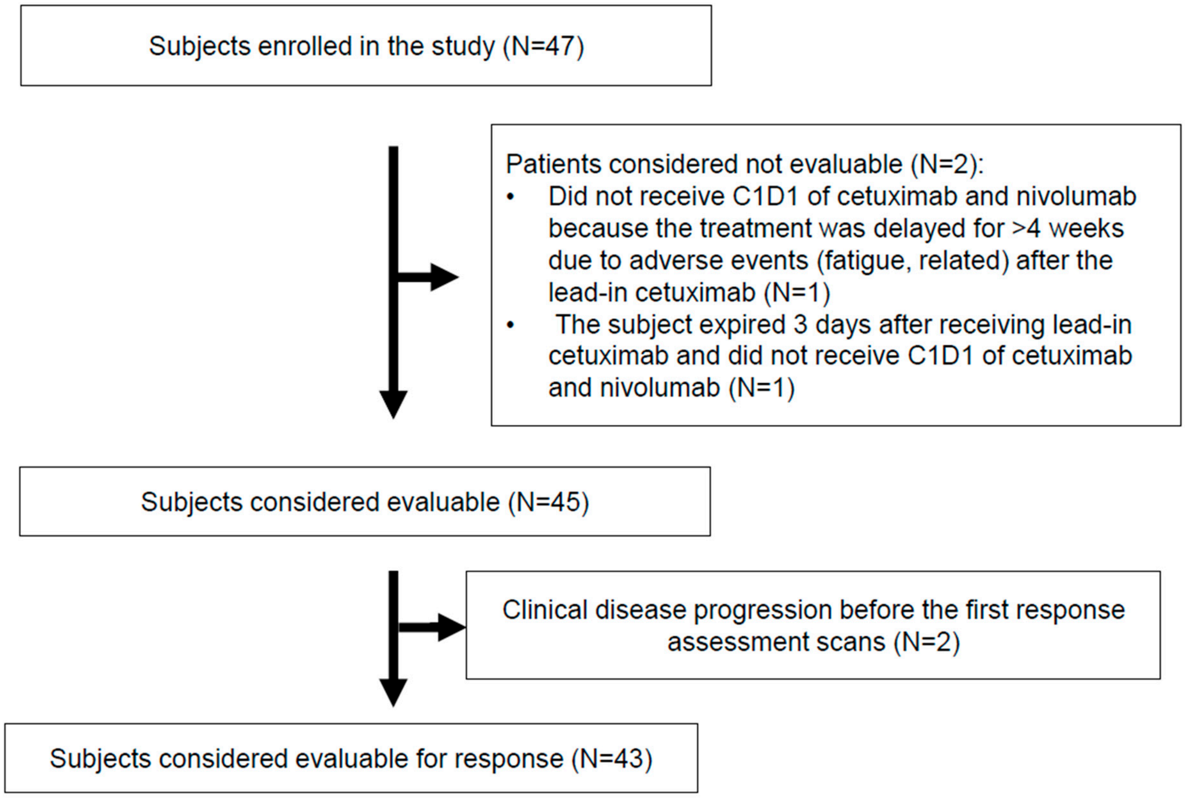

Figure S1. Consort flow diagram of the phase I/II clinical trial: concurrent cetuximab and nivolumab in patients with recurrent and/or metastatic head and neck squamous cell carcinoma.

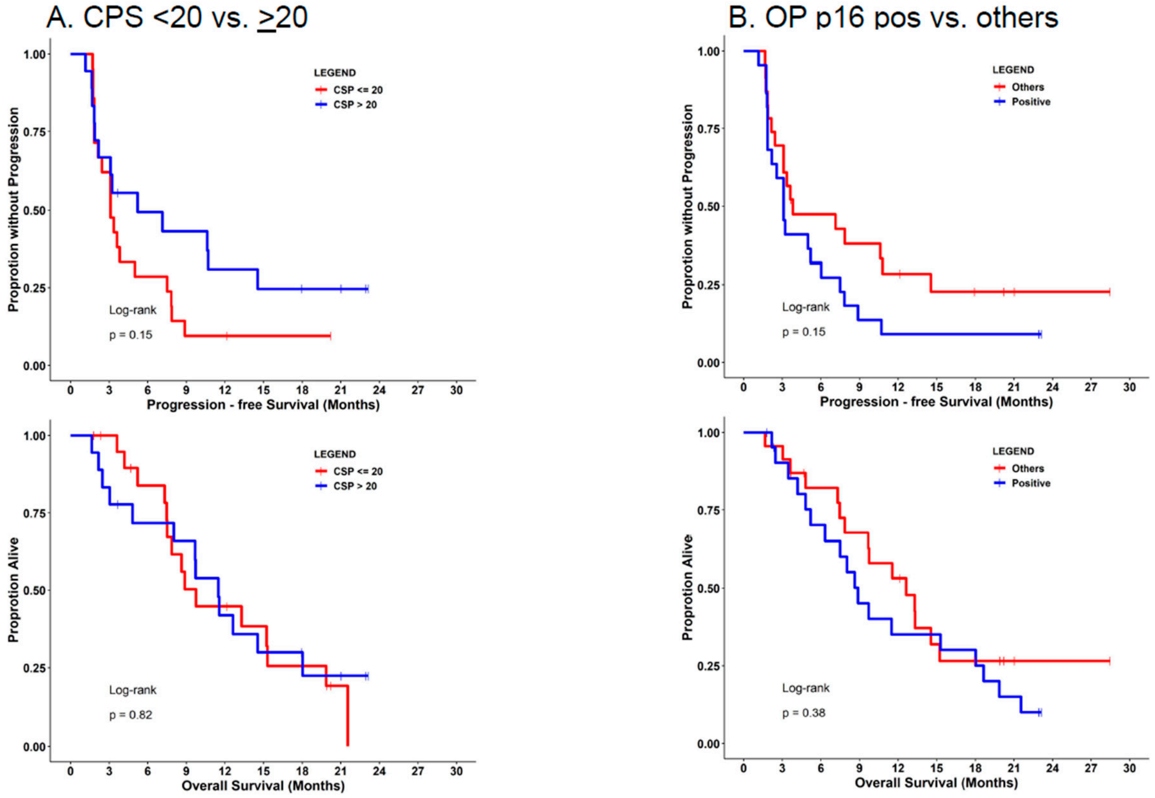

Figure S2 A) Progression-free survival (PFS) and Overall survival (OS) comparison between the tumors with combined positivity score (CPS) <20 and ≥20 in patients given combination of cetuximab and nivolumab in the clinical trial. B) PFS and OS comparison between p16-positive oropharynx and others in patients given combination of cetuximab and nivolumab in the clinical trial.

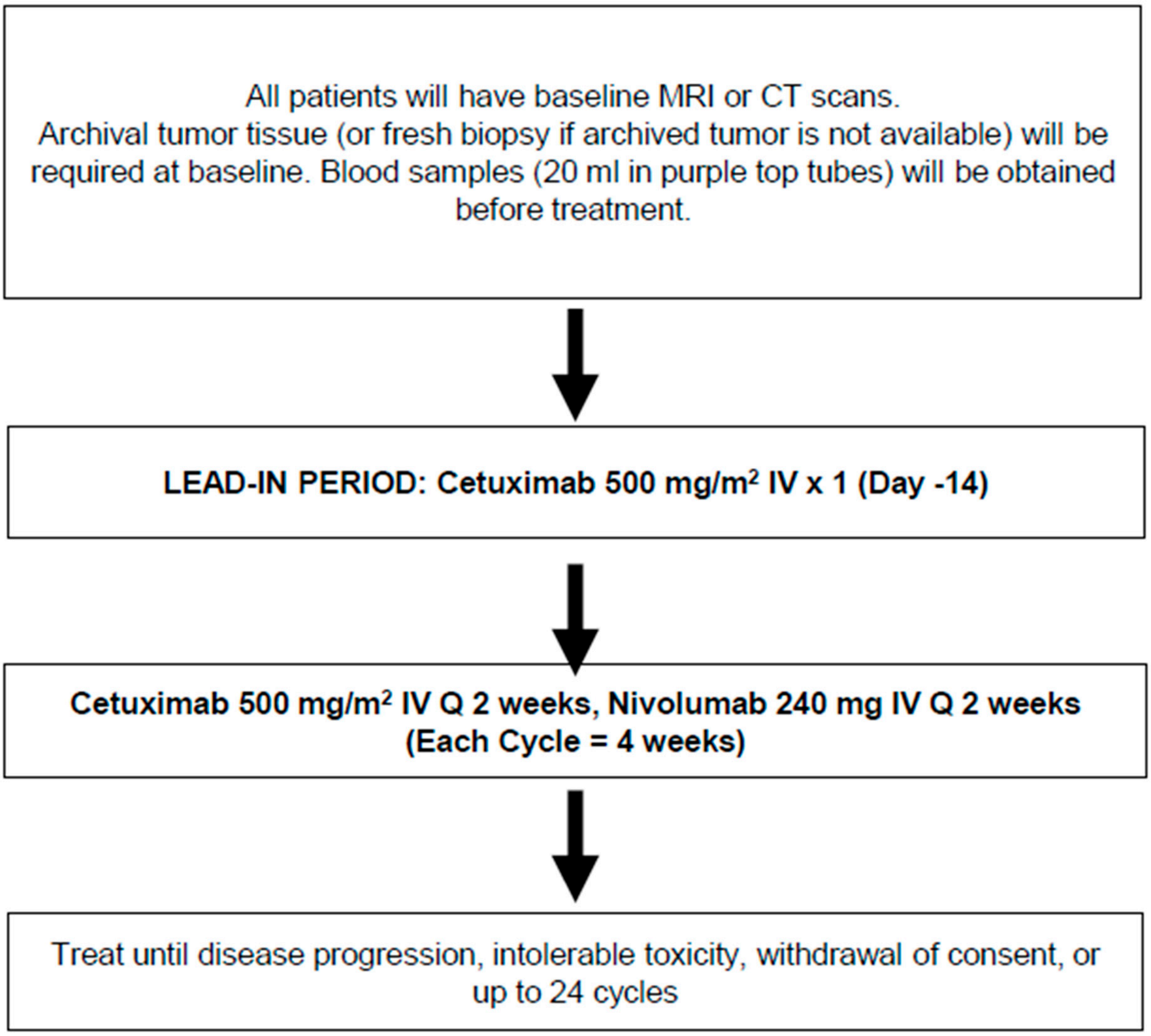

Figure S3. Study schema of the phase I/II clinical trial: concurrent cetuximab and nivolumab in patients with recurrent and/or metastatic head and neck squamous cell carcinoma.

Table S1. Description of prior platinum exposure with radiation and prior palliative Treatments before receiving cetuximab and nivolumab (N=45)

**Prior platinum exposure with radiation**		**# of patients**
Yes		36
No		9
**Line of Prior Palliative Treatments**	**Description**	**# of patients**
0	Enrolled as first line due to having platinum-refractory recurrent disease after primary chemoradiation	3
	Enrolled as first line due to having persistent disease after primary chemoradiation	7
	Enrolled as first line due to having progressed following palliative radiation for recurrent and/or metastatic disease	1
1	Platinum+5-FU+cetuximab	6
	Platinum+paclitaxel	9
	Platinum+paclitaxel+cetuximab	1
	pembrolizumab	6
	Nivolumab	6
	Nivolumab+ipilimumab	2
	Nivolumab+ipilimumab/placebo	4
2	Platinum+5-FU+cetuximab	1
	Platinum+cetuximab	1
	Platinum+paclitaxel+cetuximab	1
	pembrolizumab	4
3	pembrolizumab	1
	Docetaxel	1

Table S2. Complete Treatment-Related Adverse Events (N=45)

TOXICITY	Toxicity Category	Grade 1 (N)	Grade 2 (N)	Grade 3 (N)	Grade 4 (N)
Alanine aminotransferase increased	Investigations	-	2(4.4)	-	-
Alkaline phosphatase increased	Investigations	-	1(2.2)	1(2.2)	-
Allergic reaction	Immune system disorders	-	1(2.2)	-	-
Anemia	Blood and lymphatic system disorders	-	-	1(2.2)	-
Anorexia	Metabolism and nutrition disorders	2(4.4)	2(4.4)	-	-
Arthralgia	Musculoskeletal and connective tissue disorders	1(2.2)	-	-	-
Aspartate aminotransferase increased	Investigations	1(2.2)	1(2.2)	-	-
Bloating	Gastrointestinal disorders	-	1(2.2)	-	-
Blood bilirubin increased	Investigations	-	1(2.2)	-	-
Cardiac disorders - Other, specify	Cardiac disorders	1(2.2)	-	-	-
Chills	General disorders and administration site conditions	1(2.2)	-	-	-
Conjunctivitis	Eye disorders	-	1(2.2)	-	-
Diarrhea	Gastrointestinal disorders	4(8.9)	2(4.4)	-	-
Dizziness	Nervous system disorders	-	1(2.2)	1(2.2)	-
Dry eye	Eye disorders	1(2.2)	-	-	-
Dry skin	Skin and subcutaneous tissue disorders	6(13.3)	1(2.2)	-	-
Dysgeusia	Nervous system disorders	-	1(2.2)	-	-
Dyspnea	Respiratory, thoracic and mediastinal disorders	-	1(2.2)	1(2.2)	-
Edema face	General disorders and administration site conditions	1(2.2)	-	-	-
Eye disorders - Other, specify	Eye disorders	1(2.2)	-	-	-
Fall	Injury, poisoning and procedural complications	2(4.4)	-	-	-
Fatigue	General disorders and administration site conditions	5(11.1)	10(22.2)	6(13.3)	-
Flu like symptoms	General disorders and administration site conditions	2(4.4)	2(4.4)	-	-
General disorders and administration site conditions - Other, specify	General disorders and administration site conditions	-	-	-	1(2.2)
Generalized muscle weakness	Musculoskeletal and connective tissue disorders	-	2(4.4)	1(2.2)	-
Hypercalcemia	Metabolism and nutrition disorders	1(2.2)	-	-	-
Hyperglycemia	Metabolism and nutrition disorders	-	1(2.2)	-	-
Hypertension	Vascular disorders	-	1(2.2)	1(2.2)	-
Hyperthyroidism	Endocrine disorders	1(2.2)	-	-	-
Hypokalemia	Metabolism and nutrition disorders	1(2.2)	1(2.2)	-	-
Hypomagnesemia	Metabolism and nutrition disorders	8(17.8)	2(4.4)	1(2.2)	-
Hyponatremia	Metabolism and nutrition disorders	1(2.2)	1(2.2)	1(2.2)	-
Hypophosphatemia	Metabolism and nutrition disorders	-	3(6.7)	-	-
Hypothyroidism	Endocrine disorders	1(2.2)	3(6.7)	-	-
Infections and infestations - Other, specify	Infections and infestations	-	2(4.4)	-	-
Infusion related reaction	General disorders and administration site conditions	1(2.2)	1(2.2)	-	-

Memory impairment	Nervous system disorders	1(2.2)	-	-	-
Mucosal infection	Infections and infestations	1(2.2)	-	-	-
Mucositis oral	Gastrointestinal disorders	3(6.7)	1(2.2)	-	-
Myocarditis	Cardiac disorders	-	-	1(2.2)	-
Nail loss	Skin and subcutaneous tissue disorders	-	1(2.2)	-	-
Nausea	Gastrointestinal disorders	6(13.3)	2(4.4)	-	-
Oral dysesthesia	Gastrointestinal disorders	1(2.2)	-	-	-
Oral pain	Gastrointestinal disorders	1(2.2)	1(2.2)	-	-
Pain of skin	Skin and subcutaneous tissue disorders	1(2.2)	-	-	-
Palmar-plantar erythrodysesthesia syndrome	Skin and subcutaneous tissue disorders	2(4.4)	3(6.7)	1(2.2)	-
Papulopustular rash	Infections and infestations	5(11.1)	2(4.4)	-	-
Paronychia	Infections and infestations	7(15.6)	2(4.4)	-	-
Peripheral sensory neuropathy	Nervous system disorders	-	1(2.2)	-	-
Pleural effusion	Respiratory, thoracic and mediastinal disorders	-	1(2.2)	-	-
Pneumonitis	Respiratory, thoracic and mediastinal disorders	-	1(2.2)	-	-
Pruritus	Skin and subcutaneous tissue disorders	6(13.3)	1(2.2)	-	-
Rash acneiform	Skin and subcutaneous tissue disorders	16(35.6)	12(26.7)	2(4.4)	-
Rash maculo-papular	Skin and subcutaneous tissue disorders	1(2.2)	2(4.4)	1(2.2)	-
Sinus tachycardia	Cardiac disorders	1(2.2)	-	-	-
Skin and subcutaneous tissue disorders - Other, specify	Skin and subcutaneous tissue disorders	7(15.6)	3(6.7)	-	-
Skin infection	Infections and infestations	1(2.2)	1(2.2)	-	-
Vomiting	Gastrointestinal disorders	1(2.2)	-	-	-
Weight loss	Investigations	1(2.2)	1(2.2)	-	-
White blood cell decreased	Investigations	-	-	1(2.2)	-
Overall		5(11.1)	22(48.9)	13(28.9)	1(2.2)

Table S3. Complete Immune-Related Adverse Events (N=45)

TOXICITY	Toxicity Category	Grade 1 (N)	Grade 2 (N)	Grade 3 (N)	Grade 4 (N)
Alanine aminotransferase increased	Investigations	-	2(4.4)	-	-
Alkaline phosphatase increased	Investigations	-	1(2.2)	1(2.2)	-
Anemia	Blood and lymphatic system disorders	-	-	1(2.2)	-
Anorexia	Metabolism and nutrition disorders	1(2.2)	1(2.2)	-	-
Aspartate aminotransferase increased	Investigations	-	1(2.2)	1(2.2)	-
Bloating	Gastrointestinal disorders	-	1(2.2)	-	-
Diarrhea	Gastrointestinal disorders	2(4.4)	-	-	-
Dizziness	Nervous system disorders	-	-	1(2.2)	-
Dyspnea	Respiratory, thoracic and mediastinal disorders	1(2.2)	-	1(2.2)	-
Fall	Injury, poisoning and procedural complications	1(2.2)	-	-	-
Fatigue	General disorders and administration site conditions	3(6.7)	5(11.1)	3(6.7)	-
Generalized muscle weakness	Musculoskeletal and connective tissue disorders	-	1(2.2)	-	-
Hypercalcemia	Metabolism and nutrition disorders	1(2.2)	-	-	-
Hyperthyroidism	Endocrine disorders	1(2.2)	-	-	-
Hyponatremia	Metabolism and nutrition disorders	1(2.2)	1(2.2)	1(2.2)	-
Hypophosphatemia	Metabolism and nutrition disorders	-	1(2.2)	-	-
Hypothyroidism	Endocrine disorders	1(2.2)	5(11.1)	-	-
Hypoxia	Respiratory, thoracic and mediastinal disorders	-	1(2.2)	-	-
Memory impairment	Nervous system disorders	1(2.2)	-	-	-
Mucositis oral	Gastrointestinal disorders	-	1(2.2)	-	-
Myocardial infarction	Cardiac disorders	-	-	1(2.2)	-
Myocarditis	Cardiac disorders	-	-	1(2.2)	-
Nail loss	Skin and subcutaneous tissue disorders	-	1(2.2)	-	-
Nausea	Gastrointestinal disorders	3(6.7)	1(2.2)	-	-
Pleural effusion	Respiratory, thoracic and mediastinal disorders	-	1(2.2)	-	-
Pneumonitis	Respiratory, thoracic and mediastinal disorders	-	1(2.2)	-	-
Rash maculo-papular	Skin and subcutaneous tissue disorders	1(2.2)	1(2.2)	1(2.2)	-
Skin and subcutaneous tissue disorders - Other, specify	Skin and subcutaneous tissue disorders	1(2.2)	-	-	-
Vomiting	Gastrointestinal disorders	1(2.2)	-	-	-
Overall		5(11.1)	11(24.4)	7(15.6)	0(0.0)

Supplemental File 1: Detailed clinical trial description

TITLE: A PHASE I/II STUDY OF CONCURRENT CETUXIMAB AND NIVOLUMAB IN PATIENTS WITH RECURRENT AND/OR METASTATIC HEAD AND NECK SQUAMOUS CELL CARCINOMA

COORDINATING SITE:
Moffitt Cancer Center
Tampa, Florida 33612

PARTICIPATING SITES:
Emory University School of Medicine
Atlanta, Georgia 30322

The Ohio State University
Columbus, Ohio 43210

PRINICIPAL INVESTIGATOR (Moffitt/Coordinating Center):
Christine H. Chung, M.D.
Senior Member
Moffitt Cancer Center
12902 Magnolia Drive
Tampa, FL 33612
Phone: 813-745-5431
Fax: 813-745-7229
E-mail: Christine.Chung@Moffitt.Org

CO-PRINICIPAL INVESTIGATOR (Emory):
Nabil Saba, M.D.
Professor
Emory University School of Medicine
1365 Clifton Road, C2110
Atlanta, GA, 30322
Phone: 404-778-3126
FAX: 404-778-5676
E-mail: nfsaba@emory.edu

SITE PRINICIPAL INVESTIGATOR (Emory):
Conor Steur, M.D.
Assistant Professor
Emory University School of Medicine
1365 Clifton Road, C2110
Atlanta, GA, 30322
Phone: 404-778-1436
FAX: 404-778-5676
E-mail: csteuer@emory.edu

SITE PRINICIPAL INVESTIGATOR (The Ohio State University):
Marcelo Bonomi, M.D.
Professor
The Ohio State University
460 W 10th Ave, Columbus, OH 43210
Phone: 614-293-0463
E-mail: Marcelo.Bonomi@osumc.edu

BIOSTATISTICIAN:
Michael Schell, Ph.D.
Senior Member
Moffitt Cancer Center

12902 Magnolia Drive
Tampa, FL 33612
Phone: 813-745-6061
E-mail: Michael.Schell@Moffitt.Org

Lilly Oncology supplied agent: **Cetuximab**

Bristol-Myers Squibb supplied agent: **Nivolumab**

Funding Support: **Lilly Oncology**
The James and Esther King Biomedical Research Program

LIST OF ABBREVIATIONS

**AE**	Adverse event
**CBC**	Complete blood count
**CR**	Complete response
**CRF**	Case Report Form
**CTCAE**	Common Terminology Criteria for Adverse Events
**DLT**	Dose-limiting toxicity
**DSMP**	Data Safety Monitoring Plan
**EGFR**	Epidermal growth factor receptor
**EOT**	End of Treatment
**FDA**	Food and Drug Administration
**FFPE**	Formalin-fixed paraffin-embedded
**GCP**	Good Clinical Practice
**HIPAA**	Health Insurance Portability and Accountability Act
**HNSCC**	Head and neck squamous cell carcinoma
**HPV**	Human papillomavirus
**ICH**	International Conference on Harmonisation
**IFN-γ**	Interferon gamma
**IND**	Investigational New Drug Application
**IRB**	Investigational Review Board
**MTD**	Maximum tolerated dose
**NIH**	National Institutes of Health
**PD**	Progressive disease
**PD-1**	Programmed death 1
**PR**	Partial response
**QA**	Quality Assurance
**QC**	Quality Control
**RECIST**	Response Evaluation Criteria In Solid Tumors
**SAE**	Serious adverse event

**SD**	Stable disease
**ULN**	Upper limit of normal

PROTOCOL SYNOPSIS

**Title of study**	**A phase I/II study of concurrent cetuximab and nivolumab in patients with recurrent and/or metastatic head and neck squamous cell carcinoma**
**Investigational drugs**	Cetuximab<br>Nivolumab
**Principal Investigators**	1. Christine H. Chung, M.D. (Moffitt/Coordinating Site)<br>2. Conor Steuer, M.D. (Emory)<br>3. Marcelo Bonomi (The Ohio State University)
**Funding Organization**	Lilly Oncology
**Study Sites**	1. Moffitt Cancer Center<br>2. Emory University<br>3. The Ohio State University
**Clinical Phase**	Phase I/II
**Objectives**	**Primary Objectives:**<br>Phase I: To determine the safety and tolerability of concurrent cetuximab and nivolumab in patients with recurrent and/or metastatic HNSCC.<br><br>Phase II Cohort A: To determine the 1-year overall survival rate of concurrent cetuximab and nivolumab in patients who had progressed on at least one prior line of treatment for their recurrent and/or metastatic HNSCC.<br><br>Phase II Cohort B: To determine the 1-year overall survival rate of concurrent cetuximab and nivolumab in patients who has not had any prior treatments for their recurrent and/or metastatic HNSCC.<br><br>**Secondary Objectives:**<br>• To estimate response rate of patients treated with concurrent cetuximab and nivolumab who have recurrent and/or metastatic HNSCC.<br>• To estimate progression-free survival of patients treated with concurrent cetuximab and nivolumab who have recurrent and/or metastatic HNSCC.<br>• To evaluate the toxicity of the cetuximab and nivolumab combination in this patient population.<br><br>**Exploratory Objectives:**<br>• To identify potential biomarkers related to response to concurrent cetuximab and nivolumab in patients with recurrent and/or metastatic HNSCC.<br>• To determine a quantitative radiomics based on CT and/or PET images as a prognostic biomarker in recurrent or metastatic HNSCC.
**Study Design**	**Phase I**<br>Dose level 1: Lead-in cetuximab 500 mg/m^2 alone (Day -14 before Cycle 1 only) followed by nivolumab 240 mg IV + cetuximab 500 mg/m^2 every 2 weeks for 24 cycles or discontinuation per section 3.3.4.<br><br>Dose level -1: Lead-in cetuximab 500 mg/m^2 alone (Day -14 before Cycle 1 only) followed by nivolumab 240 mg IV + cetuximab 250 mg/m^2 every 2 weeks for 24 cycles or discontinuation per section 3.3.4.

	Each cycle is 4 weeks. Cetuximab is given alone as a lead-in period (Day -14 before Cycle 1 only). Cycle 1 and Day 1 of nivolumab and cetuximab will be given 14 days after the lead-in dose of cetuximab.<br><br>Pre-medication with steroid before cetuximab and/or nivolumab is not permitted in the trial.<br><br>Non-steroidal pre-medications are permitted.<br><br>**Phase II**<br>Dose level to be determined by the phase I results.
**Number of Patients**	**Phase I**: 3-12 patients<br>**Phase II**:<br>Cohort A: 45* patients (42 + 3 or 39 + 6)**<br><br>* 45 evaluable patients will be treated at the MTD. In order to be considered evaluable, patients must complete the lead-in period and receive the C1D1 doses of cetuximab and nivolumab. Non-evaluable subjects will be replaced.<br><br>** Patients treated at the MTD in the phase I portion of the study will be counted as a part of the phase II patient population.<br><br>Cohort B: 43 patients
**Description of Cohort A and Cohort B**	• <u>**Cohort A:**</u><br>o Patients must have recurrent or metastatic HNSCC that is not amenable to local therapy with curative intent (surgery or radiation therapy with or without chemotherapy).<br>o Patients with persistent disease following radiation therapy administered with a chemotherapy sensitizer may also be included.<br>o Patients must have progressed on at least one prior line of chemotherapy, targeted therapy, palliative radiation and/or biological therapy regimen for their recurrent and/or metastatic HNSCC. However, if patients are likely to be intolerant to standard first-line systemic chemotherapy, the patients are eligible to enroll to this study as the first-line therapy. Additionally, patients with persistent disease or platinum-refractory recurrent disease (recurs within 6 months of last dose of chemotherapy given as a sensitizer to definitive radiation) may enroll in this study as a first-line therapy.<br>o Prior treatment with a combination of cetuximab and a PD-1/PD-L1 inhibitor is excluded. Prior treatment with cetuximab or a PD-1/PD-L1 inhibitor is allowed as long as not previously given in combination.<br><br>• <u>**Cohort B:**</u><br>o Patients must have recurrent or metastatic HNSCC that is not amenable to local therapy with curative intent (surgery or radiation therapy with or without chemotherapy).<br>o Patients with persistent disease following radiation therapy administered with a chemotherapy sensitizer may also be included.<br>o Patient must **NOT** have any systemic therapy for recurrent and/or metastatic disease except if given as a part of a multimodality treatment (i.e. re-irradiation and systemic therapy for curable intent of locally recurrent disease).

	- Patients that refuse potentially curative salvage surgery for recurrent disease are ineligible. - Prior treatment with a combination of cetuximab and a PD-1/PD-L1 inhibitor is **NOT** included. - Prior treatment with a PD-1/PD-L1 inhibitor is **NOT** included. - Prior treatment with cetuximab or EGFR inhibitors given concurrently with radiation as a radiation sensitizer is included. However, cetuximab or EGFR inhibitors given in the recurrent and/or metastatic setting is **NOT** included.
**Discontinuation Criteria**	- Patients with grade 3 or 4 infusion reaction must not receive further treatment with cetuximab. - Patients with grade 3 or 4 infusion reaction must not receive further treatment with nivolumab. - Patients with grade 4 hypertension must not receive further treatment with cetuximab. - If treatment is interrupted for more than 12 consecutive weeks, patient's protocol treatment will be discontinued. - Extraordinary Medical Circumstances: If at any time the constraints of this protocol are detrimental to the patient's health, the protocol treatment should be discontinued. - Patients who develop progressive disease will be discontinued from the protocol therapy. - Patients who develop unacceptable toxicity will be discontinued from the protocol therapy. - Patients may withdraw consent and withdraw from the study at any time for any reason.

SCHEMA

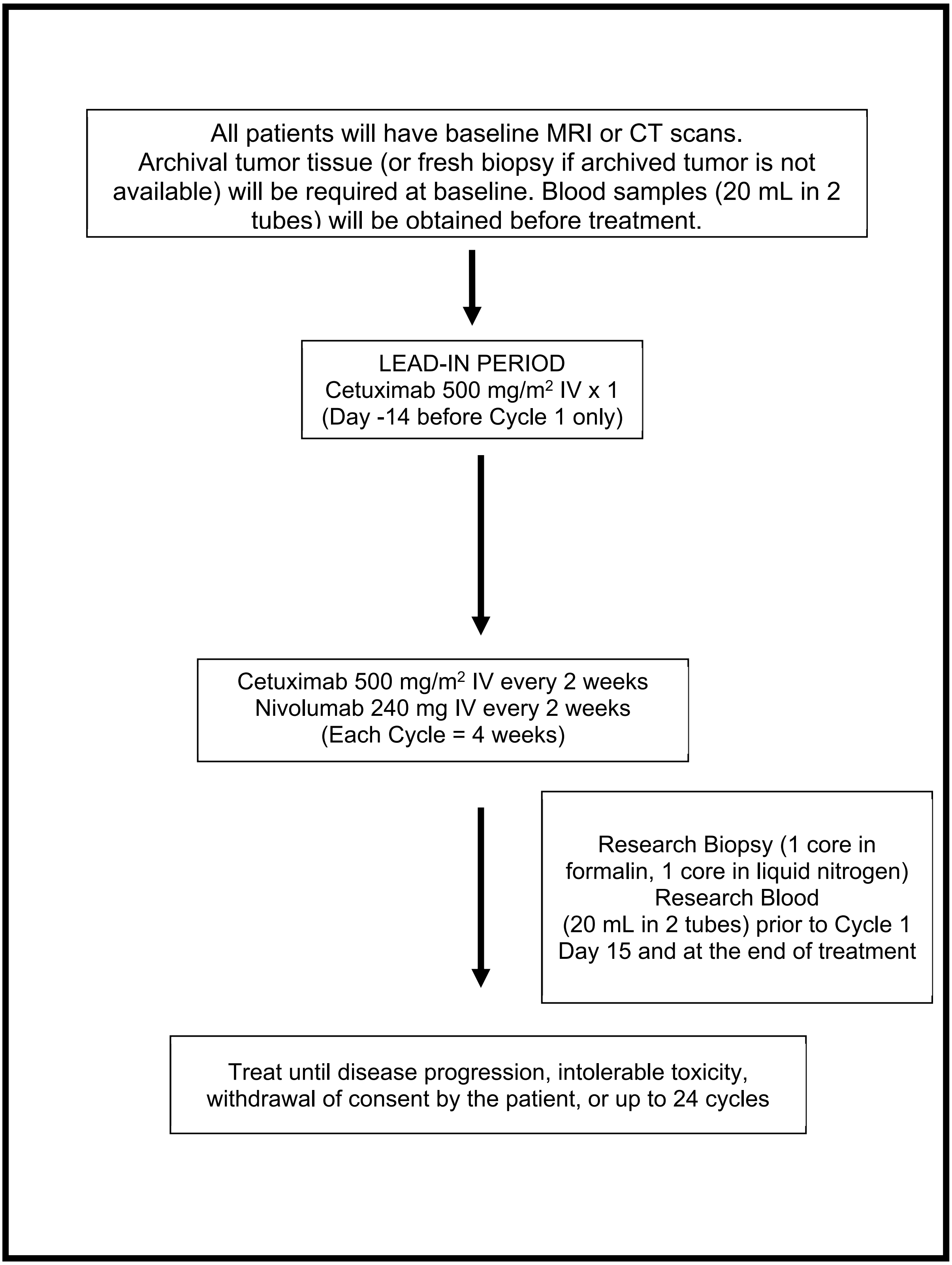
All patients will have baseline MRI or CT scans.
Archival tumor tissue (or fresh biopsy if archived tumor is not available) will be required at baseline. Blood samples (20 mL in 2 tubes) will be obtained before treatment.
LEAD-IN PERIOD
Cetuximab 500 mg/m2 IV x 1
(Day -14 before Cycle 1 only)
Cetuximab 500 mg/m2 IV every 2 weeks
Nivolumab 240 mg IV every 2 weeks
(Each Cycle = 4 weeks)
Research Biopsy (1 core in formalin, 1 core in liquid nitrogen)
Research Blood
(20 mL in 2 tubes) prior to Cycle 1 Day 15 and at the end of treatment
Treat until disease progression, intolerable toxicity, withdrawal of consent by the patient, or up to 24 cycles

1. TREATMENT STUDY CALENDER

Test	Pre-therapy (Day -42 to -15)	Lead-in period, cetuximab only (Day -14 before Cycle 1 only)	Every 2 weeks while on study drugs	End of Treatment (30 days after final dose)	Every 3 months after EOT[a]	End of Study (2 years after EOT)[a]
Window	N/A	N/A	±3 days	±3 days	±2 week	±2 week
History and physical [b]	X	X	X	X	X	X
Medical History [c]	X					
CBC and diff [d]	X	X	X	X		X
Serum chemistries [e]	X	X	X	X		X
Liver function panel [f]	X		X	X	X	X
PT, PTT, INR	X			X		
TSH	X		X[n]	X	X	X
Tissue for research purposes (paraffin embedded) [g]	X		X (Day 8 to 14 before C1D15 treatment)	X		
Blood for research purposes	X		X[k]	X		X
Toxicity assessment [h]	X	X	X	X		X
Tumor measurements [i]	X		X[l]	X[m]		
Serum B-HCG [j]	X					
Survival Follow-up				X	X	X

[a.] The first follow-up visit should be an in-clinic visit to complete the AE follow-up requirements listed in section 7.2.5. After this visit, once the patient is off treatment and has fully recovered from study drug-related toxicities or the patient enrolls in a hospice, the follow-up visits can be done by a phone call, in which case all procedures except survival follow-up may be omitted. If AE review is complete at first the first follow-up visit (e.g. subject started subsequent anticancer therapy) but SAE review is still required the visit can be limited to this review and conducted by a phone call or e-visit only.
[b.] History and physical to include ECOG performance status assessment and weight. History and physical requirement for Day 15 of each cycle is only during the phase I portion of the study. History and physical is required on only Day 1 of each cycle during the phase II portion of the study. Once the phase I portion of the study is completed (the recommended phase II dose is confirmed and approved through Moffitt's Protocol Monitoring Committee), patients enrolled at the recommended phase II dose under the phase I portion may cross over to the phase II schedule. Physical exam needs not be repeated if performed within 72 hours prior to dosing.
[c.] Relevant medical history must include smoking status (current smoker, former smoker, never smoker), number of pack years, and year of smoking cessation. Relevant medical history will also include p16 status, if known. Historic radiologic assessments will also be accessed, in accordance with protocol section 6.2, to further characterize the disease under study and to contribute to exploratory objectives.

[d] CBC and diff (complete blood count and differential) include white blood cell count, absolute neutrophil count, hematocrit, hemoglobin, platelets, % lymphocytes, % monocytes, % neutrophils, other differentials. Safety labs may be collected up to 72 hours prior to dosing.
[e] Serum chemistries include a Complete Metabolic Panel (including Na, Cl, CO2, K, BUN, creatinine, Ca) AND Mg, Phos. Safety labs may be collected up to 72 hours prior to dosing.
[f] Liver function panel includes Alk Phos, total bilirubin, SGOT (AST), SGPT (ALT), total protein. Safety labs may be collected up to 72 hours prior to dosing.
[g] If tumor paraffin blocks (or sufficient slides) are not available, fresh biopsy will be obtained before the first treatment. If fresh biopsy is indicated, one core (or punch) will be collected in formalin and the second core (or punch) will be collected in liquid nitrogen. The end of treatment optional biopsy may be performed up to 30 days after the EOT visit as scheduling may require.
[h] Toxicity assessment: Common Terminology Criteria for Adverse Events (CTCAE) version 4.1 will be used. All toxicity grades (including grade 1) should be captured on the case report forms. All toxicities that occurred during treatment and until 30 days following completion of therapy should be followed until resolution.
[i] Tumor measurement: The same type of scan should be used for repeat measurements. The scans may be performed up to 7 days before the projected corresponding treatment visit. Scans in screening will include: CT- or MRI-neck, CT-chest, CT-abdomen, and CT-pelvis. Response scans can be limited to the anatomy of areas of known and suspected disease. Scans must be performed with contrast.
[j] Pregnancy test should be done in women of childbearing age who are sexually active and may potentially be pregnant. Prior to study enrollment, women of childbearing potential must be advised of the importance of avoiding pregnancy during trial participation and the potential risk factors for an unintentional pregnancy. In addition, men enrolled on this study should understand the risks to any sexual partner of childbearing potential and should practice an effective method of birth control. All women of childbearing potential MUST have a negative pregnancy test within 7 days prior to enrollment. If the pregnancy test is positive, the patient must not receive investigational product and must not be enrolled in the study. In addition, all women of childbearing potential should be instructed to contact the Investigator immediately if they suspect they might be pregnant (e.g., missed or late menstrual period) at any time during study participation. The Investigator must immediately notify Moffitt Cancer Center and Lilly Oncology in the event of a confirmed pregnancy in a patient participating in the study.
[k] The post-treatment research blood sample after getting the combination dose of cetuximab and nivolumab on C1D1 can be collected any time from Days 8 through 15 of Cycle 1 as long as it is collected prior to any study treatment of C1D15 cetuximab and nivolumab. A second post-treatment research blood sample is due on Cycle 4 Day 1 (or 3 months from C1D1 if off treatment).
[l] Radiology assessments every 6 weeks for Cycle 1-4 (i.e. C2D15, C4D1), then every 2 Cycles during Cycle 5-6 (i.e. C6D1) and then every 3 cycles during Cycle 7-24 (i.e. C9D1, C12D1, C15D1, C18D1, C21D1, C24D1) while on study drugs, and will include: CT- or MRI-neck, CT-chest, CT-abdomen, and CT-pelvis. Scans must be performed with contrast.
m. Scans are only required at the end of treatment visit if not performed within 4 weeks prior.
n. TSH required at the start of each cycle only (i.e. not required on day 15).

2.2. TRIAL OBJECTIVES

2.2.1. Primary Objective - Phase I

The primary objective of the phase I portion of the trial is to determine the safety and tolerability of concurrent cetuximab and nivolumab in patients with recurrent and/or metastatic HNSCC.

2.2.2. Primary Objective - Phase II

Phase II Cohort A: To determine the 1-year overall survival rate of concurrent cetuximab and nivolumab in patients who had progressed on at least one prior line of treatment for their recurrent and/or metastatic HNSCC.

Phase II Cohort B: To determine the 1-year overall survival rate of concurrent cetuximab and nivolumab in patients who has not had any prior treatments for their recurrent and/or metastatic HNSCC.

2.2.3. Secondary Objectives

The secondary objectives of phase II are:

1. To estimate response rate of patients treated with concurrent cetuximab and nivolumab who have recurrent and/or metastatic HNSCC.
2. To estimate progression-free survival of patients treated with concurrent cetuximab and nivolumab who have recurrent and/or metastatic HNSCC.
3. To evaluate the toxicity of the cetuximab and nivolumab combination in this patient population.

2.2.4. Exploratory Objectives

The exploratory objectives are:

1. To identify potential biomarkers related to response to concurrent cetuximab and nivolumab in patients with recurrent and/or metastatic HNSCC.
2. To determine a quantitative radiomics based on CT and/or PET images as a prognostic biomarker in recurrent or metastatic HNSCC.

3. DESCRIPTION OF DESIGN AND STUDY POPULATION

3.1 OVERALL TRIAL DESIGN AND PLAN

This is a phase I/II prospective multicenter trial to investigate the efficacy and safety of the combination of cetuximab and nivolumab. The cetuximab 500 mg/m^2 every 2 week dosing schedule in patients with recurrent and/or metastatic HNSCC has been evaluated by a multicenter randomized prospective phase II study and has been shown to have efficacy and toxicity similar to a conventional dosing schedule of 500 mg/m^2 loading dose followed by 250 mg/m^2 every week.[1, 2]

In the phase I portion of the study, patients with recurrent and/or metastatic HNSCC will be treated at:

- Dose level 1: Lead-in cetuximab 500 mg/m^2 alone (Day -14 of Cycle 1 only) followed by nivolumab 240 mg IV + cetuximab 500 mg/m^2 every 2 weeks for 24 cycles or discontinuation per section 3.3.4.

- Dose level -1: Lead-in cetuximab 500 mg/m^2 alone (Day -14 of Cycle 1 only) followed by nivolumab 240 mg IV + cetuximab 250 mg/m^2 every 2 weeks for 24 cycles or discontinuation per section 3.3.4.

Each cycle is 4 weeks. Cetuximab is given alone in lead-in period at Day -14 of Cycle 1 only. In all subsequent doses starting on Cycle 1 Day 1, nivolumab and cetuximab will be given concurrently. Dose-limiting toxicities (DLT) will be assessed with the initiation of combination therapy (Cycle 1 Day 1) and will continue for the duration of Cycle 1 (4 weeks).

Pre-medication with steroids before cetuximab and/or nivolumab is not permitted in this trial.

Non-steroidal pre-medications are permitted, including:

- Diphenhydramine 50 mg PO or IV (or equivalent dose of antihistamine such as cetirizine).
- Acetaminophen 500-1000 mg PO (or equivalent dose of antipyretic such as paracetamol).

Dose level 1 has been confirmed as the recommended phase 2 dose, therefore the phase II dosing is dose level 1 at the start of treatment, as discussed below in section 3.2.2.

3.2. DISCUSSION OF STUDY DESIGN

3.2.1. Phase I

This is designed to determine the safety of the cetuximab and nivolumab administration. The purpose of having a lead-in period with cetuximab monotherapy (Day -14 before Cycle 1 only) is to exclude patients with cetuximab-related hypersensitivity reaction before assessing the DLT of the combination regimen.

Eligible patients will begin with a 14-day period with cetuximab alone (Day -14 before Cycle 1 only). This will be followed by subsequent doses of cetuximab and nivolumab starting on Cycle 1 Day 1 IV every 2 weeks in 28-day cycle for 24 cycles or discontinuation per section 3.3.4.

An MRI or CT of neck will be performed approximately every 6 weeks during Cycle 1-3, every 8 weeks during Cycle 5-6 and then every 12 weeks during Cycle 7-24.

3.2.2. Phase II

On April 04, 2018, an analysis of 3 evaluable patients treated at Dose Level 1 was submitted to Moffitt's Protocol Monitoring Committee, documenting that all 3 patients had completed the DLT evaluation period without experiencing any DLT's. As such, Dose Level 1 was declared the maximum tolerated dose (MTD) or the recommended phase II dose of cetuximab and accrual to the phase II portion began under Dose Level 1.

3.2.3. Number of Centers

Three sites are involved.

3.2.4. Number of Participants

Phase I: 3-12 patients
Phase II: Cohort A - 45* patients (42 + 3 or 39 + 6)**

* 45 evaluable patients will be treated at the MTD. In order to be considered evaluable, patients must complete the lead-in period and receive the C1D1 doses of cetuximab and nivolumab. Non-evaluable subjects will be replaced.

** Patients treated at the MTD in the phase I portion of the study will be counted as a part of the phase II patient population.

Phase II: Cohort B - 43 patients

* 43 evaluable patients will be treated in Cohort B. In order to be considered evaluable, patients must complete the lead-in period and receive the C1D1 doses of cetuximab and nivolumab. Non-evaluable subjects will be replaced.

3.3. SELECTION OF STUDY POPULATION

3.3.1. Main Diagnosis for Study Entry

All patients included in this trial must have recurrent and/or metastatic HNSCC.

3.3.2. Inclusion Criteria

- Patients must have histologically or cytologically confirmed squamous cell carcinoma of oral cavity, oropharynx, paranasal sinuses, nasal cavity, hypopharynx, or larynx. Squamous cell carcinoma of unknown primary in cervical lymph node can be included only if p16 status is positive.

- **Cohort A:**
  - Patients must have recurrent or metastatic HNSCC that is not amenable to local therapy with curative intent (surgery or radiation therapy with or without chemotherapy).

    - Patients with persistent disease following radiation therapy administered with a chemotherapy sensitizer may also be included.
    - Patients must have progressed on at least one prior line of chemotherapy, targeted therapy, palliative radiation and/or biological therapy regimen for their recurrent and/or metastatic HNSCC. However, if patients are likely to be intolerant to standard first-line systemic chemotherapy, the patients are eligible to enroll to this study as the first-line therapy. Additionally, patients with persistent disease or platinum-refractory recurrent disease (recurs within 6 months of last dose of chemotherapy given as a sensitizer to definitive radiation) may enroll in this study as a first-line therapy.
    - Prior treatment with a combination of cetuximab and a PD-1/PD-L1 inhibitor is excluded. Prior treatment with cetuximab or a PD-1/PD-L1 inhibitor is allowed as long as not previously given in combination.

- **<u>Cohort B:</u>**
    - Patients must have recurrent or metastatic HNSCC that is not amenable to local therapy with curative intent (surgery or radiation therapy with or without chemotherapy).
    - Patients with persistent disease following radiation therapy administered with a chemotherapy sensitizer may also be included.
    - Patient must **NOT** have any systemic therapy for recurrent and/or metastatic disease except if given as a part of a multimodality treatment (i.e. re-irradiation and systemic therapy for curable intent of locally recurrent disease).
    - Patients that refuse potentially curative salvage surgery for recurrent disease are ineligible.
    - Prior treatment with a combination of cetuximab and a PD-1/PD-L1 inhibitor is **NOT** included.
    - Prior treatment with a PD-1/PD-L1 inhibitor is **NOT** included.
    - Prior treatment with cetuximab or EGFR inhibitors given concurrently with radiation as a radiation sensitizer is included. However, cetuximab or EGFR inhibitors given in the recurrent and/or metastatic setting is **NOT** included.

- Patients must have measurable disease, defined as at least one lesion that can be accurately measured in at least one dimension (longest diameter to be recorded) as outlined in RECIST version 1.1.
- Patients must be ≥ 18 years of age.
- Life expectancy of greater than 3 months.
- ECOG performance status ≤ 2
- Patients must have normal organ function as defined below:
    - Absolute neutrophil count > 1,500/µL
    - Hemoglobin > 9 g/dL
    - Platelets > 100,000/µL
    - Total bilirubin ≤ 1.5 mg/dL X institutional upper limits of normal (ULN)
    - AST (SGOT)/ALT (SGPT) ≤ 3 X institutional ULN (or 5.0 X the ULN in the setting of liver metastasis)
    - Serum creatinine of ≤ 1.5 X ULN or creatinine clearance > 40 mL/minute (using Cockcroft/Gault formula): Female creatinine clearance = (140 - age in years) x weight in kg x 0.8572 x serum creatinine in mg/ dL; Male creatinine clearance = (140 - age in years) x weight in kg x 1.0072 x serum creatinine in mg/dL.
- Because the teratogenicity of cetuximab is not known, the patient, if sexually active, must be postmenopausal, surgically sterile, or using effective contraception (hormonal or barrier methods).
- Female patients of childbearing potential must have a negative serum pregnancy test within 7 days prior to enrollment.
- Ability to understand and the willingness to sign a written informed consent document.

3.3.3. Exclusion Criteria

- Patients who experienced grade 3 or above skin toxicity from prior EGFR inhibiting therapy.
- Patients who have experienced grade 3 or above toxicity from prior anti-PD1 therapy.

- Patients who have p16 negative squamous cell carcinoma of unknown primary in cervical lymph node.
- Patients with primary nasopharynx or salivary gland cancers are excluded.
- Patients who have had chemotherapy, biological therapy or definitive radiation within 4 weeks of the study enrollment or those who have not recovered from adverse events to ≤ Grade 1 due to agents administered more than 4 weeks earlier.
- Patients who had undergone any major surgery within 4 weeks of study enrollment.
- Patients who had undergone any palliative radiation within 2 weeks of study enrollment.
- Patients who have had other investigational agents within 4 weeks or 5 half-lives, whichever is shorter, of the study enrollment.
- Patients who have known leptomeningeal metastases or untreated or symptomatic brain metastases. Treated, asymptomatic brain metastasis can be included.
- Uncontrolled intercurrent illness including, but not limited to, ongoing or active infection, autoimmune disease requiring systemic steroids, symptomatic congestive heart failure, unstable angina pectoris, cardiac arrhythmia, or psychiatric illness/social situations that would limit compliance with study requirements.
- Subjects with a condition requiring systemic treatment with either corticosteroids (> 10 mg daily prednisone equivalents) or other immunosuppressive medications within 14 days of study drug administration. Inhaled or topical steroids and adrenal replacement doses > 10 mg daily prednisone equivalents are permitted in the absence of active autoimmune disease.
- The patient has clinically relevant coronary artery disease or history of myocardial infarction in the last 12 months or high risk of uncontrolled arrhythmia or uncontrolled cardiac insufficiency.
- The patient has uncontrolled or poorly controlled hypertension (>180 mmHg systolic or > 130 mmHg diastolic) at the time of enrollment.
- The patient has a history of allergic reactions attributed to compounds of chemical or biologic composition similar to those of cetuximab and/or nivolumab.
- The patient is pregnant or breast-feeding.
- Patients with known active HIV, Hep B, or Hep C infection will be excluded. If not clinically indicated, the patients do not need to be tested.

3.3.4. Discontinuation Criteria

Patients will be removed from study when any of the following criteria applies:

- Patients with grade 3 or 4 infusion reaction must not receive further treatment with cetuximab.
- Patients with grade 3 or 4 infusion reaction must not receive further treatment with nivolumab.
- Patients with grade 4 hypertension must not receive further treatment with cetuximab.
- If treatment is interrupted for more than 12 consecutive weeks, patient's protocol treatment will be discontinued.
- Extraordinary Medical Circumstances: If at any time the constraints of this protocol are detrimental to the patient's health, the protocol treatment should be discontinued.
- If patient develops progressive disease, then the patient will discontinue the protocol therapy.
- If patient develops unacceptable toxicity, then the patient will discontinue the protocol therapy.
- Patients may withdraw consent and withdraw from the study at any time for any reason.
- The reason and date for patient removal from the study must be documented in the Case Report Form (CRF).

3.3.5. Premature Discontinuation of the Study as a Whole

Those who discontinue protocol therapy early will be followed for response until progression and for survival for 2 years from the End of Treatment. Patients removed from study for unacceptable adverse events will be followed until resolution or stabilization of the adverse event.

4. TREATMENTS

4.1. CETUXIMAB LEAD-IN PERIOD

Cetuximab will be administered intravenously starting on Day -14 before Cycle 1 only as lead-in period. Starting dose is 500 mg/m^2 administered with acetaminophen and a histamine H1-receptor antagonist such as cetirizine. Premedication with steroid will not be allowed.

4.1.1. Definition of Cetuximab Lead-In Toxicity

Patients with grade 3 or 4 infusion reaction must not receive further treatment with cetuximab.

4.2 COMBINATION OF CETUXIMAB AND NIVOLUMAB

4.2.1. Phase I

During phase I, patients will be enrolled sequentially and treated at one of the dose levels identified below.

- Dose Level 1: Lead-in cetuximab 500 mg/m^2 alone (Day -14 before Cycle 1 only) followed by nivolumab 240 mg IV + cetuximab 500 mg/m^2 every 2 weeks for 24 cycles or discontinuation per section 3.3.4.

- Dose Level -1: Lead-in cetuximab 500 mg/m^2 alone (Day -14 before Cycle 1 only) followed by nivolumab 240 mg IV + cetuximab 250 mg/m^2 every 2 weeks for 24 cycles or discontinuation per section 3.3.4.

Each cycle is 4 weeks. Cetuximab is given alone in lead-in period at Day -14 before Cycle 1 only. In all subsequent doses starting Cycle 1 Day 1, nivolumab and cetuximab will be given concurrently. DLT assessment will be performed during Cycle 1 and will start with the initiation of the combination of cetuximab and nivolumab (4 weeks).

4.2.2. Phase II

On April 04, 2018, an analysis of 3 evaluable patients treated at Dose Level 1 was submitted to Moffitt's Protocol Monitoring Committee, documenting that all 3 patients had completed the DLT evaluation period without experiencing any DLT's. As such, Dose Level 1 was declared the maximum tolerated dose (MTD) or the recommended phase II dose of cetuximab and accrual to the phase II portion began under Dose Level 1.

4.2.3. Definition of Dose-Limiting Toxicity of Cetuximab and Nivolumab

The phase I of the study will enroll 3 to 6 patients per dose level using a standard 3+3 design. The DLT period will start on Cycle 1 Day 1 of cetuximab and nivolumab and end on Cycle 1 Day 28. The toxicity after the lead-in cetuximab (Day -14 before Cycle 1 only) prior to the dosing of cetuximab and nivolumab will not be included to the assessment of DLT (Day -14 to Day -1).

The MTD will be determined using the following de-escalation rules:

- A cohort of three patients will be entered at Dose Level 1.
- If none of these patients experiences a DLT (see below for the criteria) during Cycle 1 (4 weeks), Dose Level 1 will be the recommended dose for Phase II.
- If 1 of these 3 patients experiences a DLT, 3 more patients will be enrolled at Dose Level 1. If 1 of 6 patients at this dose level experiences a DLT, the phase II dose will be Dose Level 1. If 2 or more DLTs occur within Dose Level 1, then the MTD will have been exceeded, and 6 new patients will be enrolled at Dose Level -1.
- If ≤1 of 6 patients at Dose Level -1 experiences a DLT, the phase II dose will be Dose Level -1. If 2 or more DLTs occur within Dose Level -1, then the MTD will have been exceeded. This combination is determined to be unsafe to conduct the phase II portion of the trial and the trial will be discontinued.

The target DLT rate is <25%. The MTD will be defined as the dose of cetuximab and nivolumab in which <1 of 3 patients experience a DLT or <2 of 6 patients experience a DLT with the next higher dose having at least 2 patients experiencing a DLT. The MTD is the highest dose at which at most 1 of 6 patients has a DLT.

No dose escalations or de-escalations are permitted within each patient's treatment, although dose delays will be permitted. A patient who is withdrawn from the study before the completion of Cycle 1 for a reason other than a DLT will be replaced.

This study will utilize the Cancer Therapy Evaluation Program Common Terminology Criteria for Adverse Events (CTCAE) version 4.1 for toxicity and event reporting. DLTs will be observed until patients have completed Cycle 1 (4 weeks).

A DLT will be defined as any of the following events:

1. Grade 3 or 4 immune-related toxicities, including dermatitis, hepatitis, thyroiditis, colitis, and pneumonitis.
2. Grade 4 cetuximab-related rash.
3. Grade 3 or 4 neutropenia (i.e., absolute neutrophil count < 1000 cells/mm^3) that is associated with a fever ≥ 38.5°C or lasting longer than 5 days.
4. Grade 3 thrombocytopenia with bleeding or grade 4 thrombocytopenia.
5. Any grade 3 or 4 non-hematologic toxicity per NCI CTCAE v4.1 criteria that are probably or definitely related to study therapy, except for alopecia, nausea, and vomiting.

4.2.4. Evaluation of Response to Cetuximab and Nivolumab

During the treatment, a complete history and physical (including weight) and tumor assessment by physical examination on Day 1 of each cycle will be performed and documented. Complete blood count with differential and a comprehensive metabolic profile will be performed every 2 weeks. Radiological response will be assessed every 6 weeks by CT or MRI scans during Cycle 1-4, every 8 weeks during Cycle 5-6 and then every 12 weeks during Cycle 7-24.

4.3 DOSE LEVELS

All toxicities should be graded according to the Common Terminology Criteria for Adverse Events (version 4.1).

4.3.1. Phase I

	Cetuximab – Lead-in Day -14 before Cycle 1 only	Nivolumab – Lead-in Day -14 before Cycle 1 only	Cetuximab – Cycle 1 Day 1 and all subsequent doses Q 2 weeks	Nivolumab – Cycle 1 Day 1 and all subsequent doses Q 2 weeks
Dose Level 1	500 mg/m^2	None	500 mg/m^2	240 mg
Dose Level -1	500 mg/m^2	None	250 mg/m^2	240 mg

4.3.2. Phase II

Once the MTD or the recommended phase II dose of cetuximab is determined in phase I, accrual to phase II will begin.

4.4. CETUXIMAB DOSE MODIFICATIONS

When the medications need to be held for toxicity reasons, both cetuximab and nivolumab are always held together and restart together.

4.4.1. Dose Modifications for Cetuximab-related Immunological Toxicities

If the patient experiences any drug-related event ≤ Grade 2, the dose of cetuximab and nivolumab should be continued at the given dose level.

If the patient experiences any drug-related event ≥ Grade 3, the dose of cetuximab and nivolumab should be discontinued until resolution of the toxicities to grade 1 or baseline. Upon restarting, the physician may choose to reduce the dose of Cetuximab per the dose reduction table below.

In the event of a prolonged (≥7 consecutive days) Grade 2 drug-related toxicities, the investigator may choose to pause cetuximab and nivolumab for up to 4 weeks to allow the patient to recover followed by a dose reduction (the hold and reduction are both at the treating physician's discretion). Only the cetuximab may be dose reduced, and dose reductions are according to the following table:

	Cetuximab<br>Cycles 1-24 (Q 2 weeks)	Nivolumab<br>Cycles 1-24 (Q 2 weeks)
Dose Level 1	500 mg/m^2	240 mg
Dose Level -1	250 mg/m^2	240 mg
Dose Level -2	125 mg/m^2	240 mg

For cetuximab-related toxicity, treatment with oral/IV corticosteroids is allowed only for toxicities ≥ Grade 3.

Use of corticosteroids (≤ 10 mg daily prednisone equivalents) is allowed. Inhaled or topical steroids and adrenal replacement doses > 10 mg daily prednisone equivalents are permitted in the absence of active autoimmune disease.

4.4.2. Dose Modifications for Cetuximab-related Non-Immunological Toxicities

If the patient experiences any drug-related event ≤ Grade 2, the dose of cetuximab and nivolumab should be continued at the given dose level.

If the patient experiences any drug-related event ≥ Grade 3, the dose of cetuximab and nivolumab should be discontinued until resolution (to baseline) of the toxicities, or until stabilization. Upon resumption, the physician may choose to reduce the dose of Cetuximab per the table below. As an exception, continued treatment in the face of Grade 3 hypomagnesemia and hypophosphatemia will be allowed at the treating physician's discretion. Low magnesium is expected while treating with cetuximab and can be safely replenished as needed throughout treatment according to local guidelines.

If the patient experiences any acute onset or worsening pulmonary symptoms, the doses of cetuximab and nivolumab should be delayed. The management should follow the pulmonary toxicity management as detailed in Section 4.5 Table 2 Pneumonitis and Appendix 4 Pulmonary Adverse Event Management Algorithm because interstitial lung disease from cetuximab and pneumonitis from nivolumab cannot be reliably distinguished.

In the event of Grade ≥ 3 rash, treatment with cetuximab should be paused until recovery to Grade ≤ 2. Treatment should be resumed at a reduced dose (see Section 4.3). If Grade ≥ 3 rash does not resolve to Grade ≤ 2 within 14 days of stopping cetuximab treatment and despite optimal supportive care, the patient should not receive any further treatment with cetuximab.

In the event of a prolonged (≥7 consecutive days) Grade 2 drug-related toxicities, the investigator may choose to pause cetuximab and nivolumab for up to 4 weeks to allow the patient to recover followed by a dose reduction (the hold and reduction are both at the treating physcian's discretion). Only the cetuximab may be dose reduced, and dose reductions are according to the following table:

	Cetuximab<br>Cycles 1-24 (Q 2 weeks)	Nivolumab<br>Cycles 1-24 (Q 2 weeks)
Dose Level 1	500 mg/m^2	240 mg
Dose Level -1	250 mg/m^2	240 mg
Dose Level -2	125 mg/m^2	240 mg

4.4.3. Infusion Reactions (Cetuximab)

Treatment recommendations are provided below and may be modified based on local institutional standards, package inserts, and guidelines as appropriate.

Monitor subjects for 1 hour following cetuximab infusion on Cycle 1 Day 1 in a setting with resuscitation equipment and other agents necessary to treat anaphylaxis (eg, epinephrine, corticosteroids, intravenous antihistamines, bronchodilators, and oxygen). Monitor longer to confirm resolution of the event in subjects requiring treatment for infusion reactions. Immediately and permanently discontinue cetuximab in subjects with serious infusion reactions. Patients that have tolerated cetuximab infusions well do not require an observation period prior to starting the nivolumab infusion beyond Cycle 1 Day 1.

For Grade 1 symptoms (Mild reaction; infusion interruption not indicated; intervention not indicated):

Remain at bedside and monitor subject until recovery from symptoms. Decrease the cetuximab infusion rate by 50% and monitor closely for any worsening. The total infusion time for cetuximab should not exceed 4 hours.

For Grade 2 symptoms (Moderate reaction requires therapy or infusion interruption but responds promptly to symptomatic treatment [eg, antihistamines, non-steroidal anti-inflammatory drugs, narcotics, corticosteroids, bronchodilators, IV fluids]; prophylactic medications indicated for ≤ 24 hours):

Stop cetuximab infusion, administer bronchodilators, oxygen, etc. as medically indicated, and resume infusion at 50% of previous rate once allergic/hypersensitivity reaction has resolved or decreased to Grade 1 in severity, and monitor closely for any worsening.

For Grade 3 or Grade 4 Symptoms (Severe reaction, Grade 3: prolonged [ie, not rapidly responsive to symptomatic medication and/or brief interruption of infusion]; recurrence of symptoms following initial improvement; hospitalization indicated for other clinical sequelae
[eg, renal impairment, pulmonary infiltrates]). Grade 4: (life threatening; presser or ventilator support indicated) Immediately discontinue infusion of cetuximab and disconnect subject from tubing. Begin an IV infusion of normal saline, and treat the subject as follows. Recommend bronchodilators, epinephrine 0.2 to 1 mg of a 1:1,000 solution for subcutaneous administration or 0.1 to 0.25 mg of a 1:10,000 solution and/or diphenhydramine 50 mg IV with methylprednisolone 100 mg IV (or equivalent), as needed. Subjects have to be withdrawn immediately from the treatment and must not receive any further cetuximab treatment. Subject should be monitored until the investigator is comfortable that the symptoms will not recur. Cetuximab will be permanently discontinued.

Investigators should follow their institutional guidelines for the treatment of anaphylaxis.
Remain at bedside and monitor subject until recovery from symptoms. In the case of late occurring hypersensitivity symptoms (eg, appearance of a localized or generalized pruritus within 1 week after treatment), symptomatic treatment may be given (eg, oral antihistamine, or corticosteroids).

Once the cetuximab infusion rate has been decreased due to an allergic/hypersensitivity reaction, it will remain decreased for all subsequent infusions. If the subject has a second allergic/hypersensitivity reaction with the slower infusion rate, the infusion should be stopped and cetuximab treatment should be discontinued. If there is any question as to whether an observed reaction is an allergic/hypersensitivity reaction of Grade 1 - 4, the sponsor should be contacted immediately to discuss and grade the reaction.

4.5. NIVOLUMAB DOSE MODIFICATIONS

Note: nivolumab dose should not be modified for toxicity.

When the medications need to be held for toxicity reasons, both cetuximab and nivolumab are always held together and restart together.

Nivolumab will be held for drug-related toxicities and severe life-threatening AEs as per Table 2 below. Held doses will not be replaced. When nivolumab is restarted, resume treatment along the anticipated schedule

(i.e. doses are skipped rather than held). In the event of a treatment delay, the radiologic assessments will likewise be delayed so that restaging scans coincide with the start of the cycles specified in the study calendar.

Participants with adverse toxicity or persistent laboratory AE at grade 2 following 12 weeks of therapy may continue on the trial only if asymptomatic, controlled, and with the agreement of the principal investigator.

Table 2. Modification Guidelines for Nivolumab Drug-Related Adverse Events

Toxicity	Hold Treatment For Grade	Timing For Restarting Treatment	Discontinue Nivolumab
AST, ALT, or Increased Bilirubin	2	Toxicity resolves to grade 0-1	Toxicity does not resolve within 12 weeks of last dose
	3-4[1]	Permanently discontinue (see exceptions below)	Permanently discontinue
Diarrhea/Colitis	2-3	Toxicity resolves to grade 0-1	Toxicity does not resolve within 12 weeks of last dose or inability to reduce corticosteroid to 10 mg of prednisone or equivalent per day within 12 weeks
	4	Permanently discontinue	Permanently discontinue
Hyperthyroidism	3	Toxicity resolves to grade 0 -1	Toxicity does not resolve within 12 weeks of last dose or inability to reduce corticosteroid to 10 mg of prednisone or equivalent per day within 12 weeks
	4	Permanently discontinue	Permanently discontinue
Hypothyroidism	2-4	Therapy with nivolumab can be continued while treatment for the thyroid disorder is instituted	Therapy with nivolumab can be continued while thyroid replacement is instituted
Hypophysitis	2-3	Toxicity resolves to grade 0-1. Therapy with nivolumab can be continued while endocrine replacement therapy is instituted	Toxicity does not resolve within 12 weeks of last dose or inability to reduce corticosteroid to 10 mg of prednisone or equivalent per day within 12 weeks
	4		Permanently discontinue
Infusion Reaction	2[2]	Toxicity resolves to Grade 0-1	Permanently discontinue if toxicity develops despite

			adequate premedication
	3–4	Permanently discontinue	Permanently discontinue
Pneumonitis	2	Toxicity resolves to grade 0-1	Toxicity does not resolve within 12 weeks of last dose or inability to reduce corticosteroid to 10 mg of prednisone or equivalent per day within 12 weeks
	3-4	Permanently discontinue	Permanently discontinue
Renal Failure or Nephritis	2	Toxicity resolves to grade 0 -1	Toxicity does not resolve within 12 weeks of last dose or inability to reduce corticosteroid to 10 mg of prednisone or equivalent per day within 12 weeks
	3-4	Permanently discontinue	Permanently discontinue
All Other Drug-Related Toxicities[3.]	3	Toxicity resolves to grade 0 -1	Toxicity does not resolve within 12 weeks of last dose or inability to reduce corticosteroid to 10 mg of prednisone or equivalent per day within 12 weeks
	4	Permanently discontinue	Permanently discontinue
**Note: Permanently discontinue for any severe or Grade 3 drug-related AE that recurs or any life-threatening event.**<br>1. For patients with liver metastasis and who begin treatment with Grade 2 AST or ALT, if AST or ALT increases by greater than or equal to 50% relative to baseline and lasts for at least 1 week, then patient should be discontinued.<br>2. If symptoms are resolved within one hour of stopping drug infusion, the infusion may be restarted at 50% of the original infusion rate for the next scheduled dose.<br>3. Patients with intolerable or persistent Grade 2 drug-related AE may hold study medication at physician's discretion. Study drug should be permanently discontinued for persistent Grade 2 adverse reactions for which treatment with nivolumab has been held that do not recover to Grade 0-1 within 12 weeks of the last dose.			

4.5.1. Supportive Care Guidelines for Nivolumab

See also dose modification Section 4.5 and Table 2.

Immuno-oncology agents are associated with AEs that can differ in severity and duration than AEs caused by other therapeutic classes. Nivolumab is considered an immune-oncology agent in this protocol. Early recognition and management of AEs associated with immuno-oncology agents may mitigate severe toxicity. Management Algorithms have been developed to assist investigators in assessing and managing the following groups of AEs:

- Gastrointestinal

- Renal
- Pulmonary
- Hepatic
- Endocrinopathy
- Skin
- Neurological

The above algorithms can be found in Appendix 4

4.5.1.1. Infusion Reaction (Nivolumab)

4.5.1.1.1 Hypersensitivity infusion reactions should be reported to the sponsor within 24 hours of the event regardless of grade. The following AE terms constitute hypersensitivity infusion reactions:

- Allergic reaction
- Anaphylaxis
- Cytokine release syndrome
- Serum sickness
- Infusion reactions
- Infusion-like reactions

4.5.1.1.2 Management of infusion reactions.

Infusion reaction treatment guidelines are summarized in Table 3 below. Institutional standards for infusion reactions may be followed if the treating physician deems them acceptable for this protocol.

Table 3. Management of infusion reaction

**Grade 1**<br>Mild reaction	Increase monitoring of vital signs as medically indicated until the patient is deemed medically stable in the opinion of the investigator	None
**Grade 2**<br>Requires infusion interruption but responds promptly to symptomatic treatment (i.e., antihistamines, NSAIDS, narcotics, IV fluids); prophylactic medications indicated for ≤ 24 hours	**Stop infusion and monitor symptoms.**<br>Additional appropriate medical therapy may include but is not limited to:<br>• IV fluids<br>• Antihistamines<br>• NSAIDS<br>• Acetaminophen<br>• Narcotics<br>Increase monitoring of vital signs as medically indicated until the patient is deemed medically stable in the opinion of the investigator.<br>If symptoms resolve within one hour of stopping drug infusion, the infusion may be restarted at 50% of the original infusion rate.<br>Otherwise, dosing will be held until symptoms resolve, and the patient should be premedicated for the next scheduled dose.<br>Patients who develop Grade 2 toxicity despite adequate premedication should be permanently discontinued from further trial treatment administration.	Subject may be premedicated 1.5h (± 30 minutes) prior to infusion of nivolumab with:<br>• Diphenhydramine 50 mg PO (or equivalent dose of antihistamine)<br>• Acetaminophen 500-1000 mg PO (or equivalent dose of antipyretic)
**Grade 3 or 4**<br>**Grade 3**:<br>Prolonged (i.e., not rapidly responsive to symptomatic medications and/or	**Stop infusion.**<br>Additional appropriate medical therapy may include but is not limited to:<br>• IV fluids<br>• Antihistamines<br>• NSAIDS	No subsequent dosing

brief interruption of infusion); recurrence of symptoms following initial improvement; hospitalization indicated for other clinical sequelae (e.g., renal impairment, pulmonary infiltrates)<br><br>**Grade 4**: Life-threatening; pressor or ventilatory support indicated	• Acetaminophen<br>• Narcotics<br>• Oxygen<br>• Pressors<br>• Corticosteroids<br>• Epinephrine<br>Increase monitoring of vital signs as medically indicated until the patient is deemed medically stable in the opinion of the investigator.<br>Hospitalization may be indicated.<br>Patient is permanently discontinued from further trial treatment administration.	

4.6 CONCOMITANT TREATMENTS

4.6.1 Steroids

Treatment with oral/IV corticosteroids (>10mg daily prednisone equivalent) is not allowed for non-immunological toxicities. Treatment with oral/IV corticosteroids (>10mg daily prednisone equivalent) for immunological toxicities is permitted as detailed in the dose modification sections.

Use of corticosteroids (≤ 10 mg daily prednisone equivalents) is allowed. Inhaled or topical steroids and adrenal replacement doses > 10 mg daily prednisone equivalents are permitted in the absence of active autoimmune disease.

4.6.2 Radiation

Palliative radiation therapy is allowed to non-target lesions at the discretion of the treating physician. As concurrent radiotherapy and nivolumab/cetuximab have not been formally evaluated, in cases where palliative radiotherapy is required for a tumor lesion, then nivolumab/cetuximab should be withheld for at least 1 week before, during, and 1 week after radiation. Subjects should be closely monitored for any potential toxicity during and after receiving radiotherapy,

6. ASSESSMENT OF EFFICACY

6.1. RECIST CRITERIA

To assess objective response, it is necessary to estimate the overall tumor burden at baseline to which subsequent measurements will be compared. Measurable disease is defined by the presence of at least one measurable lesion.

All measurements should be recorded in metric notation in centimeters. The same method of assessment and the same technique should be used to characterize each identified lesion at baseline and during follow-up. In cases where lesions are followed by clinical measurements, radiologic assessments should continue in accordance with the schedule of events to fully characterize the lesion(s). All baseline evaluations should be performed as closely as possible to the beginning of treatment and never more than 4 weeks before registration.

At baseline, tumor lesions will be characterized as either measurable or non-measurable.

6.1.1. Measureable Lesions

Measureable lesions should be selected on the basis of their size (those with the longest diameters) and their suitability for accurate repeated measurements. The sum of the longest diameters of all measurable lesions and shortest diameters of all measurable malignant lymph nodes will be calculated at baseline and reported as the baseline sum of longest diameters. The sum longest diameter will be used to characterize the objective overall tumor response. For lesions measurable in 2 or 3 dimensions, always report the longest diameter for tumors and the axis perpendicular to the longest axis for lymph nodes at the time of each assessment.

- Complete Response (CR): The disappearance of all measurable lesions.
- Partial Response (PR): At least a 30% decrease in the sum of the longest diameters of measureable lesions, taking as reference the baseline sum longest diameter.
- Progressive Disease (PD): At least a 20% increase in the sum of the longest diameters of target lesions, taking as reference the smallest sum longest diameter recorded since the baseline measurements, or the appearance of one or more new lesion(s).
- Stable Disease (SD): Neither sufficient shrinkage to qualify for partial response nor sufficient increase to qualify for progressive disease. To be assigned a status of stable disease, measurements must have met the stable disease criteria at least once after study entry at a minimum interval of 6 weeks.

6.1.2. Non-Measureable Lesions

Non-Measurable Lesions are all other lesions or sites of disease. Measurements of these lesions are not required, but the presence or absence of each should be noted throughout follow-up.

- Complete Response (CR): The disappearance of all non-measureable lesions.
- Incomplete Response/Stable Disease (SD): The persistence of one or more non-measureable lesion(s). To be assigned a status of stable disease, measurements must have met the stable disease criteria at least once after study entry at a minimum interval of 8 weeks.
- Progressive Disease (PD): The appearance of one or more new lesion(s) and/or unequivocal progression of existing non-measureable lesions.

6.1.2. Notes on progression and new lesions

1. For equivocal findings of progression (e.g. very small and uncertain new lesions; cystic changes or necrosis in existing lesions), treatment may continue until the next scheduled assessment. If at the next scheduled assessment, progression is confirmed, the date of progression should be the earlier date when progression was suspected.
2. A previous abnormal target lymph node that became normal and subsequently enlarged in size meeting the criteria for a pathologic and measurable lymph node (a short axis of ≥1.5 cm) should be added to the sum of diameters to determine if criteria for progression are met based on target lesions.
3. A previously abnormal non-target lymph node that became normal and subsequently recurred must meet the criteria for progression based on non-target lesions to be considered progression.
4. A normal lymph node at baseline (<1.0 cm) that subsequently becomes pathologic is considered a new lesion and should be considered progression.
5. If newly abnormal lymph node(s) of unclear etiology is driving the progression event, continuation of treatment/follow-up and confirmation by a subsequent exam should be contemplated. If it becomes clear that the new lymph node has not resolved, is not resolving, or has increased in size, the date of progression would be the date the new lymph node was first documented as meeting pathologic criteria.

6.2. CENTRAL RADIOLOGY REVIEW

Baseline and response assessment radiology images will be evaluated along with historic images (CT, PET/CT, MRI collected from the time of diagnosis) for all evaluable patients enrolled at Moffitt Cancer Center. For patients who were enrolled at outside of Moffitt, baseline and response assessment radiology images from patients with response will be submitted to Moffitt for central review to confirm response. For this purpose, the scans obtained at the time of best response will also need submitted. Please reference the Imaging Data Transmittal Form for additional submission details.

7. ASSESSMENT OF SAFETY

7.1. ENDPOINTS OF SAFETY

Safety of cetuximab and nivolumab will be evaluated as indicated by intensity and incidence of adverse events, graded according to US NCI CTCAE Version 4.1. Safety endpoints include:

- Events leading to dose reduction
- Events leading to permanent treatment discontinuation
- The overall incidence and CTCAE v4.1 criteria grade of adverse events, as well as relatedness of adverse events to treatment
- Causes of death

7.2. DEFINITIONS OF ADVERSE EVENTS

7.2.1. Adverse Event

An adverse event (AE) is defined as any untoward medical occurrence, including an exacerbation of a pre-existing condition, in a patient in a clinical investigation who received a pharmaceutical product. The event does not necessarily have to have a causal relation with this treatment. Lab abnormalities not deemed clinically significant will not be collected.

7.2.2. Serious Adverse Event

A serious adverse event (SAE) is defined as any AE that results in death, is immediately life-threatening, results in persistent or significant disability/incapacity, requires or prolongs patient hospitalization, is a congenital anomaly/birth defect, or is to be deemed serious for any other reason if it is an important medical event when based on appropriate medical judgement that may jeopardize the patient and may require medical or surgical intervention to prevent one of the other outcomes listed in the above definitions.

In addition, all reports of spontaneous abortion, abuse, and/or drug dependency shall be considered as SAEs for regulatory reporting purposes.

Patients may be hospitalized for administrative or social reasons during the study (e.g., days on which infusion takes place, long distance from home to site). These and other hospitalizations planned at the beginning of the study do not need to be reported as an SAE in case they have been reported at screening visit in the source data and have been performed as planned.

Any SAEs that occur during the screening period or after lead-in cetuximab monotherapy (Day -14 to Day -1 of Cycle 1) prior to the first dose of the cetuximab and nivolumab combination do not need to be reported.

7.2.3. Intensity of Adverse Event

The intensity of adverse events should be classified and recorded according to the Common Terminology Criteria for Adverse Events (CTCAE) version 4.1 in the CRF.

7.2.4. Causal Relationship of Adverse Event

- Medical judgment should be used to determine the relationship, considering all relevant factors, including pattern of reaction, temporal relationship, de-challenge or re-challenge, confounding factors such as concomitant medication, concomitant diseases and relevant history. Assessment of causal relationship should be recorded in the CRFs.

  Yes: There is a reasonable causal relationship between the investigational product administered and the AE.

  No: There is no reasonable causal relationship between the investigational product administered and the AE.

- Worsening of the underlying disease or of other pre-existing conditions will be recorded as an (S)AE in the CRF.

- Changes in vital signs, ECG, physical examination, and laboratory test results will be recorded as an (S)AE in the CRF, if they are judged clinically relevant by the investigator.

7.2.5. Adverse Event and Serious Adverse Event Reporting

- Upon inclusion into the study, the patient's condition is assessed (e.g., documentation of history/concomitant diagnoses and diseases) and relevant changes from baseline are noted subsequently.

- All adverse events, serious and non-serious, occurring during the screening period or after lead-in cetuximab monotherapy (Day -14 to Day -1 of Cycle 1) prior to the first dose of the cetuximab and nivolumab combination do not need to be reported.

- All adverse events, serious and non-serious, occurring during the course of the combination of cetuximab and nivolumab treatment (i.e., from Cycle 1 Day 1 onward through the 100-day follow-up period) will be collected, documented, and reported to the sponsor by the investigator on the appropriate CRFs/SAE reporting forms.

- All adverse events will be followed for a minimum of 100 days after protocol treatment, except in cases where a study participant has started a new antineoplastic therapy. However, any SAE occurring after the start of a new treatment that is suspected to be related to study treatment by the investigator will be reported. To allow for this collection, the first Q3 month follow-up visit after the End of Treatment visit should be an in-clinic visit if possible for the patient to come to the clinic.

- Reporting will be done according to the specific definitions and instructions detailed in the "Adverse Event Reporting" section of the Investigator Site File.

- For each adverse event, the investigator will provide the onset date, end date, grade according to CTCAE v4.1, treatment required, outcome, seriousness, and action taken with the investigational drug. The investigator will determine the relationship of the investigational drug to all AEs as defined in Section 7.2.1.

- Adverse events with onset within first administration of cetuximab therapy and 100 days after last administration of cetuximab will be considered as on treatment. All AEs, including those persisting after end of study treatment must be followed up until they have resolved or have been sufficiently characterized or the principal investigator decides to not further pursue them.

- Serious and non-serious adverse events occurring later than 100 days after last administration of trial drugs will only be reported in case they are considered drug-related or trial (procedure) related.

- Deaths (unless they are considered drug-related or trial related) will not be reported as SAE when they occur later than 100 days after last administration of the trial.

- Although adverse events attributed to the underlying malignancy under study will be captured, disease progression itself will not be reported as an adverse event.

7.2.6. Responsibilities for SAE Reporting

The clinical management system being used for this study is The Online Collaborative Research Environment (OnCore). OnCore will be used to record all study related information for all registered subjects, including AEs and SAEs as defined in section 7.2.5. All serious adverse events (SAE) must be reported to Lilly Global Patient Safety via fax within 24 hours with a causality assessment, at 866-644-1697 or 317-453-3402. In addition, all serious adverse events (SAE) must be reported to Brystol Myers Squibb within 24 hours with a causality assessment, either via fax to 609-818-3804 or via email to Worldwide.Safety@BMS.com.

9. INVESTIGATIONAL PLAN

9.1. VISIT SCHEDULE

Details of study procedures are in the Study Calendar.

9.1.1. Screening Period

<u>Therapeutic Parameters</u>

Pre-study scans used to assess all measurable or non-measurable sites of disease must be done within **4 weeks** prior to registration.

Pre-study complete blood count (with differential and platelet count) should be done ≤ **4 weeks** before registration.

All required pre-study chemistries should be done ≤ **4 weeks** before registration.

9.1.2. End of Study Treatment and Follow-up Period

Patients will be followed for 2 years from End of Treatment. Patient will be followed by treating physicians as per standard of care.

9.1.3. Duration of Therapy

Patients will receive protocol therapy unless:

- Patients completed the 24 cycles of the treatments.
- Treatment is interrupted for more than 12 consecutive weeks; patient’s protocol treatment will be discontinued.
- Extraordinary medical circumstances have occurred. If at any time the constraints of this protocol are detrimental to the patient's health, protocol treatment should be discontinued.
- Patient develops progressive disease; then the patient will discontinue protocol therapy.
- Patient develops unacceptable toxicity; then the patient will discontinue protocol therapy.
- Patients may withdraw consent and withdraw from the study at any time for any reason.

9.1.4. Duration of Follow-up

For this protocol, all patients, including those who discontinue protocol therapy early, will be followed for response until progression and for survival for 2 years from the End of Treatment. Patients that discontinue protocol therapy early without documented disease progression may have their scans performed per standard of care. All patients must also be followed through completion of all protocol therapy. Patients removed from study for unacceptable adverse events will be followed until resolution or stabilization of the adverse event.

10. STATISTICAL METHODS

10.1. STUDY DESIGN AND SAMPLE SIZE JUSTIFICATION

10.1.1. Phase I Study

Primary Objectives:
Phase I: To determine the safety and tolerability of concurrent cetuximab and nivolumab in patients with recurrent and/or metastatic HNSCC.

Design and Sample Size: The dose escalation phase of the study will enroll 3 to 6 patients per dose level using a standard 3+3 design.

The MTD will be determined using the following de-escalation rules:

- Patients will be enrolled sequentially and treated at one of the dose levels identified below after the one dose of lead-in cetuximab. An initial set of 3 patients will be enrolled at Dose Level 1. If no DLT (see below for the criteria) is observed during Cycle 1 (4 weeks), then enrollment in the phase II as Dose Level 1 will be initiated.

- If DLT is observed in 1 patient during Cycle 1 (4 weeks), then an additional 3 patients will be enrolled at Dose Level 1 (total 6 patients). If DLT is observed in ≤ 1 of 6 patients, then enrollment in the phase II as Dose Level 1 will be initiated.

- If DLT is observed in 2 or more of 6 patients at Dose Level 1, then the MTD will have been exceeded, and a set of 6 new patients will be enrolled at Dose Level -1. If DLT is observed in ≤ 1 of 6 patients, then enrollment in the phase II as Dose Level -1 will be initiated.

- If ≥ 2 of 6 patients experience DLT at Dose Level -1, then the MTD will have been exceeded. This combination will be determined to be unsafe to conduct the phase II portion of the trial, and the trial will be discontinued.

	Cetuximab: Lead-In Day -14 of Cycle 1 only	Nivolumab: Lead-In Day -14 of Cycle 1 only	Cetuximab: Starting Cycle 1 Day 1, every 2 weeks	Nivolumab: Starting Cycle 1 Day 1, every 2 weeks
Dose Level 1	500 mg/m^2	None	500 mg/m^2	240 mg
Dose Level -1	500 mg/m^2	None	250 mg/m^2	240 mg

The DLT period will start on Cycle 1 Day 1 of cetuximab and nivolumab and end on Cycle 1 Day 28. The toxicity after the lead-in cetuximab (Day -14 before Cycle 1 only) prior to the dosing of cetuximab and nivolumab will not be included to the assessment of DLT.

The target DLT rate is <25%. The MTD will be defined as the dose of cetuximab and nivolumab in which <1 of 3 patients experience a DLT or <2 of 6 patients experience a DLT with the next higher dose having

at least 2 patients experiencing a DLT. The MTD is the highest dose at which at most 1 of 6 patients has a DLT.

No dose escalations or de-escalations are permitted within each patient's treatment regimen, although dose delays will be permitted. A patient who is withdrawn from the study before the completion of the first cycle for a reason other than a DLT will be replaced.

This study will utilize the Cancer Therapy Evaluation Program CTCAE version 4.1 for toxicity and event reporting. Dose-limiting toxicities will be observed until patients have completed Cycle 1 (4 weeks).

A dose-limiting toxicity will be defined as any of the following events:

1. Grade 3 or 4 immune-related toxicities, including dermatitis, hepatitis, thyroiditis, colitis, and pneumonitis.
2. Grade 4 cetuximab-related rash.
3. Grade 3 or 4 neutropenia (i.e., absolute neutrophil count < 1000 cells/mm^3) that is associated with a fever ≥ 38.5°C or lasting longer than 5 days.
4. Grade 3 thrombocytopenia with bleeding or grade 4 thrombocytopenia.
5. Any grade 3 or 4 non-hematologic toxicity per NCI CTCAE v4.1 criteria that are probably or definitely related to study therapy, except for alopecia, nausea, and vomiting.

10.1.2. Phase II Study

Primary Objectives:

Phase II Cohort A: To test if the overall survival rate of concurrent cetuximab and nivolumab in patients who had progressed on at least one prior line of treatment for their recurrent and/or metastatic HNSCC exceeds that of the historical data, which has an estimated one-year survival rate of 36%.

<u>Cohort A:</u> We will assume 36% one-year overall survival based on the historical data from nivolumab monotherapy.[3] With our expected 56% one-year overall survival after the combined treatment with both cetuximab and nivolumab, we will need to enroll 45 patients to reach 90% power to see a statistically significant difference (P ≤ 0.05) in overall survival. 45 evaluable patients will be treated at the MTD. In order to be considered evaluable, patients must complete the lead-in period and receive the C1D1 doses of cetuximab and nivolumab. Non-evaluable subjects will be replaced.

Phase II Cohort B: To test if the 1-year overall survival rate of concurrent cetuximab and nivolumab in patients who has not had any prior treatments for their recurrent and/or metastatic HNSCC exceeds that of the historical data, which has an estimated one-year survival rate of 46%.

<u>Cohort B:</u> We will assume 46% one-year overall survival based on the historical data from pembrolizumab monotherapy.[4] With our expected 66% one-year overall survival after the combined treatment with both cetuximab and nivolumab, we will need to enroll 43 patients to reach 90% power to see a statistically significant difference (P ≤ 0.05) in overall survival. 43 evaluable patients will be treated at the MTD. In order to be considered evaluable, patients must complete the lead-in period and receive the C1D1 doses of cetuximab and nivolumab. Non-evaluable subjects will be replaced.

The sample size was obtained for the one-sample log-rank test from PASS15, with the assumption that accrual will take one year with one-year follow-up.

Secondary Objectives:

- To estimate response rate of patients treated with concurrent cetuximab and nivolumab who have recurrent and/or metastatic HNSCC.

The response rate will be estimated using binomial theory with Wilson's method for the 95% confidence interval.

- To estimate progression-free survival of patients treated with concurrent cetuximab and nivolumab who have recurrent and/or metastatic HNSCC.

The median and 1-year progression-free survival rates will be estimated from the Kaplan-Meier curve with its 95% confidence interval.

- To evaluate the toxicity of the cetuximab and nivolumab combination in this patient population.

The toxicity data will be provided in a table by major toxicity category and for the highest toxicity grade for the patient.

Exploratory Objectives:

- To identify potential biomarkers related to response to concurrent cetuximab and nivolumab in patients with recurrent and/or metastatic HNSCC.

Exploratory analysis to assess the association of potential biomarkers with disease response will be made using logistic regression.

- To determine a quantitative radiomics based on CT and/or PET images as a prognostic biomarker in recurrent or metastatic HNSCC

Exploratory analysis to assess the association of potential radiomics signatures with disease response will be made using logistic regression.

11. ADMINISTRATION, HANDLING OF DATA, AND SAFETY MONITORING

11.1. PROTOCOL AMENDMENTS

Any changes to the protocol will be made in the form of an amendment and must be approved by the coordinating site IRB before implementation. Any modifications made to the protocol or informed consent document according to local requirements or any other reason may also require approval from sponsoring agencies.

11.2. INFORMED CONSENTS

An investigator will explain to each participant the nature of the study, its purpose, procedures involved, expected duration, and potential risks and benefits. All patients will be informed that participation in the study is voluntary and that they may withdraw from the study at any time, and that withdrawal of consent will not affect their subsequent medical treatment. This informed consent will be given by means of a standard written statement and will be submitted for IRB approval prior to use. No patient will enter the study before informed consent has been obtained. In accordance with the Health Information Portability and Accountability Act (HIPAA), the written informed consent document (or a separate document to be given in conjunction with the consent document) will include a subject authorization to release medical information to the study sponsor and supporting agencies and/or allow these bodies, a regulatory authority, or Institutional Review Board access to the patient's medical information, which includes all hospital records relevant to the study, including the patient's medical history.

11.3. ETHICS AND GOOD CLINICAL PRACTICE

This study must be carried out in compliance with the protocol and Good Clinical Practice, as described in:

1. ICH Harmonized Tripartite Guidelines for Good Clinical Practice 1996.
2. US 21 Code of Federal Regulations dealing with clinical studies (including parts 50 and 56

concerning informed consent and IRB regulations).

3. Declaration of Helsinki, concerning medical research in humans (Recommendations Guiding Physicians in Biomedical Research Involving Human Subjects, Helsinki 1964, amended Tokyo 1975, Venice 1983, Hong Kong 1989, Somerset West 1996).

The investigator agrees to adhere to the instructions and procedures described in it and thereby to adhere to the principles of Good Clinical Practice that it conforms to.

11.4. REGULATORY AUTHORITIES

11.4.1. Institutional Review Board

Information regarding study conduct and progress will be reported to the Institutional Review Board (IRB) per the current institutional standards of each participating center.

11.4.2. Food and Drug Administration

This trial involves an Investigational New Drug (IND) exemption.

11.5. DATA QUALITY ASSURANCE

11.5.1. Data Management

All information will be collected on study-specific CRFs by the study staff at each institution. The necessary forms will be provided to each site by the Coordinating Center.

The completed forms will be forwarded to the Coordinating Center for central review and inclusion in the study dataset with relevant source documentation as outlined in the CRFs. The data submission schedule is as follows:

At the time of registration:

- Registration Form
- Informed Consent Form (signed by the patient)
- Eligibility Checklist
- Source documents related to eligibility.

Within 2 weeks after registration:

- Baseline study CRFs
- Pertinent source documents

Within 2 weeks after 30-day follow-up:

- On-study CRFs
- Pertinent source documents

All study data will be reviewed for completeness and accuracy by the Protocol Chair. The Principal Investigator (or his/her designee) at each respective institution is responsible for review and for ensuring the completeness and accuracy of the data generated by his/her institution. The study data will also be periodically reviewed by the Moffitt Cancer Center Clinical Research Office.

Registration Procedures with Moffitt Clinical Research Network (MCRN) Office

All subjects must be registered with the MCRN Coordinating Center to be able to participate in a trial. The participating site must fax or email the completed study specific eligibility checklist and registration forms, supporting documents and signed informed consent to the Coordinating Center. Unsigned or incomplete forms will be returned to the site. Once documents are received, the MCRN Research Coordinator will review them to confirm eligibility and to complete the registration process. If eligibility cannot be confirmed, the research coordinator will query the site for clarification or additional documents

as needed. Subjects failing to meet all study eligibility requirements will not be registered and will be unable to participate in the trial.

Upon completion of registration, the MCRN Research Coordinator will provide the participating site with the study sequence number on the completed registration form (**APPENDIX 3**).

It is the responsibility of the participating Investigator or designee to inform the subject of the research treatment plan and to conduct the study in compliance with the protocol as agreed upon with Moffitt Cancer Center and approved by the site's IRB.

To register a patient send the completed signed eligibility checklist along with supporting documentation to the MCRN via email at ESC_Partnerships@moffitt.or g or via fax at 813-745-5666, Monday through Friday between 8:00AM and 5:00PM (EST).

11.5.2. Meetings and Conference Calls

Scheduled meetings and conference calls will take place as needed with the medical oncology co-investigators and study personnel involved at the coordinating center and participating sites. In addition, separate meetings will be scheduled and include the protocol principal investigator, study coordinator(s), data manager(s), collaborators, and biostatistician involved with the conduct of the protocol. During these meetings, matters related to the following will be discussed: safety of protocol participants, validity and integrity of the data, enrollment rate relative to expectation, characteristics of participants, retention of participants, adherence to protocol (potential or real protocol violations), data completeness, and progress of data for objectives.

11.5.3. Monitoring and Auditing

Data will be captured in OnCore, Moffitt's Clinical Trials Database. Regulatory documents and case report forms will be monitored internally according to Moffitt Cancer Center Monitoring Policies. Monitoring will be performed regularly to verify data is accurate, complete, and verifiable from source documents; and the conduct of the trial is in compliance with the currently approved protocol/amendments, Good Clinical Practice (GCP), and applicable regulatory requirements.

To obtain access to OnCore, the External Site Coordination (ESC) office Coordinator will supply forms required to be completed by the site staff. Once the completed forms are received, the site coordinator will receive DUO access, logon/password, and information on how to access OnCore. The ESC office will provide OnCore training to the site once initial access is granted and on an ongoing basis, as needed.

Protocol Monitoring Committee (PMC)
The PMC meets monthly and reviews accrual, patterns and frequencies of all adverse events, protocol violations and when applicable, internal audit results.

The Protocol Chair is responsible for monitoring the study. Data must be reviewed to ensure the validity of data, as well as the safety of the participants. The Protocol Chair will also monitor the progress of the trial, review safety reports, and clinical trial efficacy endpoints and to confirm that the safety outcomes favor continuation of the study.

The Protocol Chair will be responsible for maintaining the clinical protocol, reporting adverse events, ensuring that consent is obtained and documented, reporting of unexpected outcomes, and reporting the status of the trial in the continuing renewal report submitted to the IRB and to the trial monitoring review group. Content of the continuing renewal report at a minimum should include year-to-date and full trial data on the following: accrual and eligibility, protocol compliance, treatment administration, toxicity and ADR reports, response, survival, regulatory compliance, and compliance with prearranged statistical goals. The report should be submitted in a timely manner according to the schedule defined by the Moffitt Cancer

Center Institutional Review Board.

Regulatory documents and case report forms will be monitored internally according to Moffitt Cancer Center Monitoring Policies. Monitoring will be performed regularly by the MCC Clinical Monitoring Core for accuracy, completeness, and source verification of data entry, validation of appropriate informed consent process, reporting of SAEs, and adherence to the protocol, Good Clinical Practice (GCP) guidelines, and applicable regulatory requirements.

12. COORDINATING CENTER AND SITE RESPONSIBILITIES

12.1. PROTOCOL CHAIR

The Protocol Chair is responsible for performing the following tasks:

- Coordinating, developing, submitting, and obtaining approval for the protocol as well as its subsequent amendments.
- Ensuring that all participating institutions are using the correct version of the protocol.
- Taking responsibility for the overall conduct of the study at all participating institutions and for monitoring the progress of the study.
- Reviewing and ensuring reporting of Serious Adverse Events (SAE).
- Reviewing data from all sites.
- Protocol chair has the authority to stop the study.

12.2. COORDINATING CENTER

The Coordinating Center is responsible for performing the following tasks:

- Ensuring that IRB approval has been obtained at each participating site prior to the first patient registration at that site and maintaining copies of IRB approvals from each site.
- Managing central patient registration.
- Collecting and compiling data from each site.
- Establishing procedures for documentation, reporting, and submitting of AEs and SAEs to the Protocol Chair and all applicable parties.
- Facilitating audits by securing selected source documents and research records from participating sites for audit, or by auditing at participating sites.

12.3. PARTICIPATING SITES

Participating sites are responsible for performing the following tasks:

- Following the protocol as written and the guidelines of Good Clinical Practice.
- Submitting data to the Coordinating Center.
- Registering all patients with the Coordinating Center by submitting patient registration form and signed informed consent promptly.
- Providing sufficient experienced clinical and administrative staff and adequate facilities and equipment to conduct a collaborative trial according to the protocol.
- Maintaining regulatory binders on site and providing copies of all required documents to the Coordinating Center.
- Collecting and submitting data according to the schedule specified by the protocol.
- Site principal investigators have the authority to stop the study at each site.

- Site principal investigators will review and ensure reporting of SAEs.

Additional responsibilities for participating sites are described below.

12.3.1 Staffing

The participating sites will provide experienced staff and adequate equipment and facilities to support this clinical trial. The participating sites will also be responsible for research staff training, human patient research, and HIPAA compliance, as well as the continuing education in these areas as required by local institutional standards.

12.3.2. Documentation

Each participating site is responsible for submitting copies of all relevant regulatory documentation to the Coordinating Center. The required documents include, but are not limited to the following: local IRB approvals (i.e., protocol, consent form, amendments, patient brochures and recruitment material), IRB membership rosters, summary of unanticipated problems or protocol deviations, and documentation of expertise of the investigators. The Coordinating Center will provide each participating site with a comprehensive list of the necessary documents. It is the responsibility of the participating sites to maintain copies of all documentation submitted to the Coordinating Center.

The requirements for data management, submissions, and monitoring are outlined below.

12.3.3. Confidentiality

All unpublished information that the Coordinating Center gives to the investigator shall be kept confidential and shall not be published or disclosed to a third party without the prior written consent of the Protocol Chair (or her designee).

12.3.4. Record Retention

Following closure of the study, each participating center will maintain a copy of all site study records in a safe and secure location. The Coordinating Center will inform the investigator at each site at such time that the records may be destroyed. This will also follow each institution guidelines

12.3.5. Publication

It is understood that any manuscript or releases resulting from the collaborative research will be circulated to all participating sites prior to submission for publication or presentation. The Protocol Chair will be the final arbiter of the manuscript content.

12.3.6. Additional Information

Each participating site is responsible for submitting additional information as requested by the Protocol Chair (or her designee).

12.4. RECORDS

Case Report Forms (CRFs) for individual patients will be provided by the Principal Investigator.

12.4.1. Source Documents

Source documents provide evidence for the existence of the patient and substantiate the integrity of the data collected. Source documents are filed at the investigator's site.

Data entered in the CRFs that are transcribed from source documents must be consistent with the source documents or the discrepancies must be explained. The investigator may need to request previous medical records or transfer records, depending on the study; also current medical records must be available.

For CRFs, all data must be derived from source documents.

12.4.2. Direct Access to Source Data and Documents

The investigator/institution will permit study-related monitoring, audits, IRB review, and regulatory inspection, providing direct access to all related source data/documents. CRFs and all source documents, including progress notes and copies of laboratory and medical test results, must be available at all times for review by the clinical study monitor and auditor and for inspection by health authorities (e.g., FDA). The

Clinical Research Associate/on-site monitor and auditor may review all CRFs and written informed consents. Data will be captured in Oncore, Moffitt's Clinical Trials Database.

12.5. STATEMENT OF CONFIDENTIALITY

Individual patient medical information obtained as a result of this study is considered confidential and disclosure to third parties is prohibited with the exceptions noted below.

Patient confidentiality will be ensured by using patient identification code numbers.

Treatment data may be given to the patient's personal physician or to other appropriate medical personnel responsible for the patient's welfare. Data generated as a result of the study need to be available for inspection on request by the participating physicians or the Grantor's representatives, by the IRB, and by the regulatory authorities.

12.6. COMPLETION OF STUDY

The IRB/competent authority needs to be notified about the end of the trial (last patient/patient out, unless specified differently in the clinical study protocol) or early termination of the trial.

13. REFERENCES

1. Fury, M.G., et al., *A randomized phase II study of cetuximab every 2 weeks at either 500 or 750 mg/m2 for patients with recurrent or metastatic head and neck squamous cell cancer.* J Natl Compr Canc Netw, 2012. **10**(11): p. 1391-8.
2. Vermorken, J.B., et al., *Open-label, uncontrolled, multicenter phase II study to evaluate the efficacy and toxicity of cetuximab as a single agent in patients with recurrent and/or metastatic squamous cell carcinoma of the head and neck who failed to respond to platinum-based therapy.* J Clin Oncol, 2007. **25**(16): p. 2171-7.
3. Ferris, R.L., et al., *Nivolumab for Recurrent Squamous-Cell Carcinoma of the Head and Neck.* N Engl J Med, 2016.
4. Burtness, B., et al., *KEYNOTE-048: Phase 3 study of first-line pembrolizumab (P) for recurrent/metastatic head and neck squamous cell carcinoma (R/M HNSCC).* ESMO 2018 Congress, 2018.
